# Anti-cancer effect of palmatine through inhibition of the PI3K/AKT pathway in canine mammary gland tumor CMT-U27 cells

**DOI:** 10.1186/s12917-023-03782-2

**Published:** 2023-10-25

**Authors:** Min-Jae Yoo, Jawun Choi, Ye-ji Jang, Sang-Youel Park, Jae-Won Seol

**Affiliations:** College of Veterinary Medicine, Jeonbuk National University, Iksan, Jeollabuk-Do 54596 Republic of Korea

**Keywords:** Canine mammary gland tumors, CMTs, Canine, Cancer, Palmatine, PI3K/AKT pathway, PTEN, PI3K inhibitor

## Abstract

Canine mammary gland tumors (CMTs) are the most common and lethal cancers in female dogs. Dysregulated phosphoinositide 3-kinases (PI3K)/AKT pathway reportedly was involved in the growth and metastasis of CMTs. However, there are few studies on therapeutic strategies for targeting the PI3K pathway in CMTs. In this study, we aimed to determine whether palmatine, a natural isoquinoline alkaloid with anti-cancer properties, could inhibit the growth of CMTs and whether the inhibitory effect was mediated through the PI3K/AKT pathway. Our in vitro experiments on CMT-U27, a CMT cell line, showed that palmatine reduced cell proliferation and induced cell death. Western blotting results revealed that palmatine decreased the protein expression of PI3K, PTEN, AKT, and mechanistic target of rapamycin in the PI3K/AKT pathway, which was supported by the results of immunocytochemistry. Additionally, palmatine suppressed the migration and tube formation of canine aortic endothelial cells as well as the migration of CMT U27 cells. Our in vivo results showed that palmatine inhibited tumor growth in a CMT-U27 mouse xenograft model. We observed a decreased expression of proteins in the PI3K/AKT pathway in tumor tissues, similar to the in vitro results. Furthermore, palmatine significantly disrupted the tumor vasculature and inhibited metastasis to adjacent lymph nodes. In conclusion, our findings demonstrate that palmatine exerts anti-cancer effects against CMTs by inhibiting PI3K/AKT signaling pathway, suggesting that palmatine has potential as a canine-specific PI3K inhibitor for the treatment of CMTs.

**Supplementary Information:**

The online version contains supplementary material available at 10.1186/s12917-023-03782-2.

## Introduction

Canine mammary gland tumors (CMTs) are the second most common tumor in dogs. They are most common in female dogs, accounting for more than 40% of the cases, and 50% are malignant [1–4]. Surgery is the common treatment for CMTs, especially for non-metastatic tumors. Dogs with benign tumors and 50% with malignant tumors can be treated with surgery alone [5]. However, the remaining 50% are impossible to operate on because the tumors are excessively large and metastatic [6]. Several chemotherapeutic agents for humans, such as doxorubicin and 5-fluorouracil, have traditionally been used to treat canine patients with inoperable CMTs [7]. However, when applied to dogs, these anti-cancer drugs have not shown significant effectiveness in the treatment of tumors [8, 9]. Despite these uncertainties, chemotherapy remains a major therapeutic option for dogs with CMTs [10], which have a poor prognosis, high recurrence rate, and short survival times after surgery [11, 12]. Therefore, there is an urgent need to identify effective canine-specific therapeutic agents for CMTs.

The phosphoinositide 3-kinases (PI3K)/AKT pathway, consisting of PI3K, phosphatase and tensin homologue (PTEN), AKT (known as protein kinase B), and the mechanistic target of rapamycin (mTOR), is one of the most important pathways that regulates cell death, proliferation, motility, metabolism, and tumor vascular growth and maintenance, known as angiogenesis [13, 14]. The key enzyme in this pathway is PI3K, which regulates the expression of sub-signals by phosphorylating phosphatidylinositol 4,5-bisphosphate (PIP2) into phosphatidylinositol (3,4,5)-trisphosphate (PIP3) [15]. Moreover, mutations in *PI3KCA*, a PI3K gene, increase downstream signaling pathways and promote tumor growth in various tumors [16–19]. Therefore, PI3K is considered an important therapeutic target, and research on PI3K inhibitors has been actively conducted over the last decade. This research has led to the discovery of PI3K inhibitors, such as alpelisib, which have been approved by the FDA and are used for treatment, showing effectiveness in humans [20]. Interestingly, recent studies have revealed similarities between human breast cancer (HBC) and CMTs in several areas, such as estrogen receptors and p53, suggesting that CMTs are an appropriate model for studying HBC [21–24]. Furthermore, PI3K mutations occur in approximately 40% of all cases in both HBC and CMTs, and the hotspot is the H1047R amino acid [20, 25]. Despite their obvious similarities, there are differences in the mechanisms underlying CMTs and HBC. For example, unlike HBC, the relationship between the clinical benefit of HER2 amplification and HER2 overexpression is not clear in CMTs [26, 27]. Thus, there is no guarantee that the currently used human-targeted PI3K inhibitors will have the same effect in canines; therefore, efforts are needed to discover canine-specific PI3K inhibitors.

Palmatine is a naturally occurring isoquinoline alkaloid derived from *Tinospora cordifolia*, *Corydalis yanhusuo*, and *Phellodendron amurense* [28–30]. It has been registered as an anti-inflammatory drug in the Chinese Pharmacopoeia and has been used to treat inflammatory disorders, gastrointestinal infections, gynecological inflammation, and liver diseases [31, 32]. Interest in palmatine has grown over the past few years and ongoing research has revealed its neuroprotective, antibacterial, antiviral, and anti-cancer properties, demonstrating its potential as a therapeutic agent against a variety of diseases [33, 34]. In studies related to its anti-cancer effect, palmatine showed considerable growth inhibition in several human cancer cell lines, such as the HepG2 human liver cancer cell line, CEM T lymphoblast cell line, K III and Lewis lung carcinoma cell lines, and MCF-7 human breast cancer cell line [35, 36]. In addition, it exhibited an anti-proliferative effect in human ER + /HER2- breast cancer cell lines, regardless of the tumor type, and suppressed metastasis to the lungs of the 4T1 triple-negative breast cancer cell line [37, 38]. Moreover, it induced cell death by inhibiting the PI3K/AKT pathway in human acute myeloid leukemia cell lines [39]. Although palmatine has proven effective in humans, few studies have been conducted in canines. Therefore, if palmatine exerts anti-cancer effects on CMTs as a PI3K inhibitor, as previously shown in humans, it could be used as a canine-specific inhibitor for the treatment of CMTs.

In this study, we demonstrated that palmatine exerts an anti-cancer effect on CMT-U27 by performing the in vitro assays including cell viability assay, migration assay, western blotting, and immunofluorescence assay. Furthermore, we validated these effects in the in vivo experiments using CMT nude mouse xenograft models. Our findings elucidated the mechanisms of the inhibitory effect of palmatine on tumor development. In detail, palmatine regulates the PI3K/AKT pathway, which plays a crucial role in tumor cell proliferation. This regulation was confirmed in both in vitro and in vivo experiments. In addition, palmatine inhibited angiogenesis, a key requirement in tumor growth and metastasis. The results of tube formation assay on canine aortic endothelial cells (CnAOECs) and immunofluorescence analysis on tumors demonstrated the reduction in both the formation of new blood vessels and the number of pre-existing tumor vasculature during palmatine treatment. Additionally, by analyzing immunofluorescent images of lymph nodes adjacent to the tumor in the xenograft models, we confirmed that palmatine reduced metastasis. In conclusion, this study provides evidence demonstrating the anti-proliferative, anti-angiogenic, and anti-metastatic properties of palmatine against CMTs by targeting the PI3K/AKT pathway. These effects are similar to those of PI3K inhibitors, suggesting that palmatine has the potential to be used effectively as a PI3K inhibitor for the treatment of CMTs.

## Materials and methods

### Cell culture and reagents

CMT-U27 cells were purchased from the American Type Culture Collection (Manassas, VA, USA) and cultured in Roswell Park Memorial Institute 1640 medium (HyClone, Logan, UT, USA) supplemented with 10% fetal bovine serum (FBS; Atlas Biologicals, Fort Collins, CO, USA). Penicillin (100 unit/mL) and streptomycin (100 μg) were purchased from Sigma-Aldrich (St. Louis, MO, USA). CnAOECs and their growth medium were purchased from Cell Applications, Inc. (San Diego, CA, USA). All the cells were incubated at 37ºC in 5% CO_2_. Palmatine and dimethyl sulfoxide (DMSO) were purchased from Sigma-Aldrich. Palmatine was prepared as a stock solution at concentrations of 200 mM in DMSO for in vitro experiments and at a concentration of 100 mg/mL in DMSO for in vivo experiments*.*

### Tumor model and palmatine treatment

Nude mice (BALB/c Slc-nu/nu, 6 weeks old) were obtained from the Central Lab, Animal lnc. (Seoul, Korea). To create a xenograft model, a small incision was made between the fourth nipple and midline of the mice using scissors. Suspensions of CMT-U27 cells (5 × 10^5^ cells in 50 μl phsphate-buffered saline (PBS)) were then injected into the fourth mammary fat pad. Two weeks after cell injection, the mice were divided into two groups: palmatine-treated (*n* = 3) and untreated (*n* = 3). The treated group received intraperitoneal injections of 50 mg/kg palmatine in PBS daily for 21 days. The untreated group was injected with the same amount of DMSO in PBS. Tumor volume was measured every two days using the following formula: length × width × width × 0.5 [40]. The mice were sacrificed by cervical dislocation after being anesthetized with CO_2_ gas, and tissues were harvested for further experiments.

### 3-(4,5-dimethylthiazol-2-yl)-5-(3-carboxymethoxyphenyl)-2-(4-sulfophenyl)-2H-tetrazolium (MTS) assay

To evaluate the effect of palmatine on cell viability, we performed a 3-(4,5-dimethylthiazol-2-yl)-5-(3-carboxymethoxyphenyl)-2-(4-sulfophenyl)-2H-tetrazolium (MTS) assay using the CellTiter 96® AQueous One Solution Cell Proliferation Assay (Promega Corporation, Wisconsin, USA). CMT-U27 cells were seeded at 8 × 10^3^ cells in a 96-well plate with 100 μl of medium and incubated at 37ºC for 24 h. Then, palmatine was treated for 18 h at each concentration. Following treatment, 20 μl of CellTiter 96® AQueous One Solution Reagent was added to each well and incubated for 2 h at 37ºC. Absorbance was measured at 490 nm using a microplate reader (Spectramax M2; Molecular Devices, CA, USA).

### Lactate Dehydrogenase (LDH) release assay

We measured the levels of lactate dehydrogenase (LDH) released from damaged CMT-U27 cells using the CytoTox 96® Non-Radioactive Cytotoxicity Assay (Promega Corporation, Wisconsin, USA). CMT-U27 cells were cultured at a density of 15 × 10^4^ cells/well in 24-well plates, incubated for 24 h, and then treated with palmatine for 18 h. Subsequently, 50 μl of supernatant and 50 μl of CytoTox 96® Reagent were loaded to each well in a 96-well plate and incubated for 30 min at room temperature in the dark room. The absorbance was measured at 570 nm using a microplate reader (Molecular Devices).

### Annexin-V / Propidium Iodide (PI) staining

Cell death was evaluated by flow cytometry using an Annexin V assay (Santa Cruz Biotechnology, Inc., Dallas, TX, USA) according to the manufacturer’s protocol. Annexin V content was estimated by measuring fluorescence at 488 nm (excitation) and 525 nm (emission) using the Guava easyCyte HT system (Millipore, Billerica, MA, USA).

### Western blotting

CMT-U27 cells were homogenized in cold lysis buffer containing a protease inhibitor cocktail (Sigma-Aldrich). Proteins were separated using sodium dodecyl sulfate–polyacrylamide gel electrophoresis and transferred onto nitrocellulose membranes. The membranes were blocked by 5% skim milk and incubated with the following primary antibodies in a blocking buffer overnight at 4ºC: Rabbit polyclonal anti-AKT (9272; Cell Signaling Technology, Inc., Beverly, MA, USA), rabbit polyclonal anti-p-AKT (9271; Cell Signaling), rabbit monoclonal anti-mTOR (2983; Cell Signaling), rabbit monoclonal anti-p-mTOR (5536; Cell Signaling), Rabbit polyclonal anti-p-PI3K (AF3242; Affinity biosciences, Cincinnati, OH, USA), rabbit polyclonal anti-PTEN (bs0686-R; Bioss, Inc., Beijing, China), mouse monoclonal anti-p-PTEN (sc-377573; Santacruz Biotechnology, CA, USA), and mouse monoclonal anti-β-actin (A5441; Sigma-Aldrich). The membranes were then incubated with horseradish peroxidase (HRP)-conjugated secondary antibodies for 1 h at room temperature. The chemiluminescent signals were enhanced using an HRP substrate (Millipore) and detected using a Fusion FX7 acquisition system (Vilbert Lourmat, Eberhardzell, Germany). The density of each band was measured using the software Quantity One (version 4.6.6) and normalized to β-actin. The relative intensity was expressed as compared to the control.

### Immunocytochemistry

The CMT-U27 cells were cultured on coverslips coated with 1% gelatin. Cells were fixed with 2% paraformaldehyde for 20 min at 4ºC and permeabilized with 0.5% Triton X-100 in PBS for 10 min. The cells were blocked with 5% animal serum (donkey or goat) in 2% bovine serum albumin in PBS for 1 h at room temperature. Subsequently, the cells were incubated with primary antibodies against anti-p-PI3K (Affinity Biosciences) and p-PTEN (Santa Cruz Biotechnology) in blocking solution at 4ºC overnight. Then, the cells were incubated with Dylight® 488 conjugated goat anti-rabbit IgG (Bethyl Laboratories) and Cy3-conjugated donkey anti-mouse IgG (Jackson ImmunoReasearch). Nuclei were stained with 4',6-Diamidino-2-Phenylindole (DAPI). The cells were then mounted with mounting medium (Dako, Carpinteria, CA, USA), and images were captured using a confocal microscope (Carl Zeiss, Inc., Jena, Germany). We used the software ImageJ (version 1.52a) to measure the mean fluorescence intensity of each channel in three different regions. The relative fluorescence intensity was represented as compared to the control.

### In vitro migration assay

CMT-U27 cells and CnAOECs were cultured in 6-well plates. After reaching 100% confluence, the cells were scratched manually using a sterile 1000-μl pipette tip. Subsequently, the culture medium was replaced with 1 ml of fresh medium containing 1% FBS, and different concentrations of palmatine were added to each well. At the indicated times after treatment, images were captured using a microscope (Nikon Eclipse TS100; Nikon Corporation, Tokyo, Japan).

### In vitro tube formation assay

Matrigel™ (Corning Inc., New York, NY, USA) was thawed overnight at 4ºC for the tube formation assay. The Matrigel was dispensed into a 24-well plate and incubated at 37ºC for 60 min for solidification. CnAOECs were seeded at a density of 1.2 × 10^5^ cells/well in fresh medium with or without palmatine and incubated at 37ºC for 2 h. Tube formation of CnAOECs was photographed under a microscope (Nikon Corporation) and quantified by counting the number of tubes from three different sections.

### Immunohistochemistry

For frozen block immunohistochemistry, tumor tissues and inguinal lymph nodes were fixed in 4% paraformaldehyde for 4 h and dehydrated overnight in 20% sucrose solution. The tissues were then embedded in a tissue-freezing medium (Leica, Wetzlar, Germany). Frozen blocks were sectioned at 20 μm, and samples were blocked with 5% goat serum in 0.03% Triton X-100 in PBS. Samples were then stained overnight at 4ºC with the following primary antibodies: Armenian hamster monoclonal anti-CD31 (MAB1398Z; Millipore), mouse monoclonal FITC-conjugated anti-α-SMA (F3777; Sigma-Aldrich), mouse multiclonal anti-pan-cytokeratin (ab961; Abcam, Cambridge, MA, USA), and rabbit polyclonal anti-LYVE-1 (11–034; AngioBio, Del Mar, CA, USA). After primary antibody staining, samples were incubated for 2 h at room temperature with the following secondary antibodies: Cy-3 conjugated goat anti-hamster IgG, Cy-3 conjugated donkey anti-mouse IgG (both from Jackson ImmunoResearch), and Dylight® 488 conjugated goat anti-rabbit IgG (Bethyl Laboratories). The nuclei were stained with DAPI. The samples were then mounted with mounting medium (Dako), and images were captured using a confocal microscope (Carl Zeiss). The mean fluorescence intensity for each antibody was measured with ImageJ software on three different images. The value was represented as the percent intensity by total fluorescence intensity.

### Statistical analysis

All data are presented as mean ± standard deviation (SD). Statistical significance between groups was determined using the unpaired Student’s *t*-test. One-way analysis of variance (ANOVA), followed by Bonferroni post hoc tests, was conducted to determine significant differences among multiple groups. Statistical analyses were performed using the GraphPad Prism software. Statistical significance was set at *p* < 0.05, * *p* < 0.05, ** *p* < 0.01, and *** *p* < 0.001.

## Results

### Palmatine inhibits tumor growth in CMT-U27 cell-derived xenograft (CDX) model

**Fig. 1:**
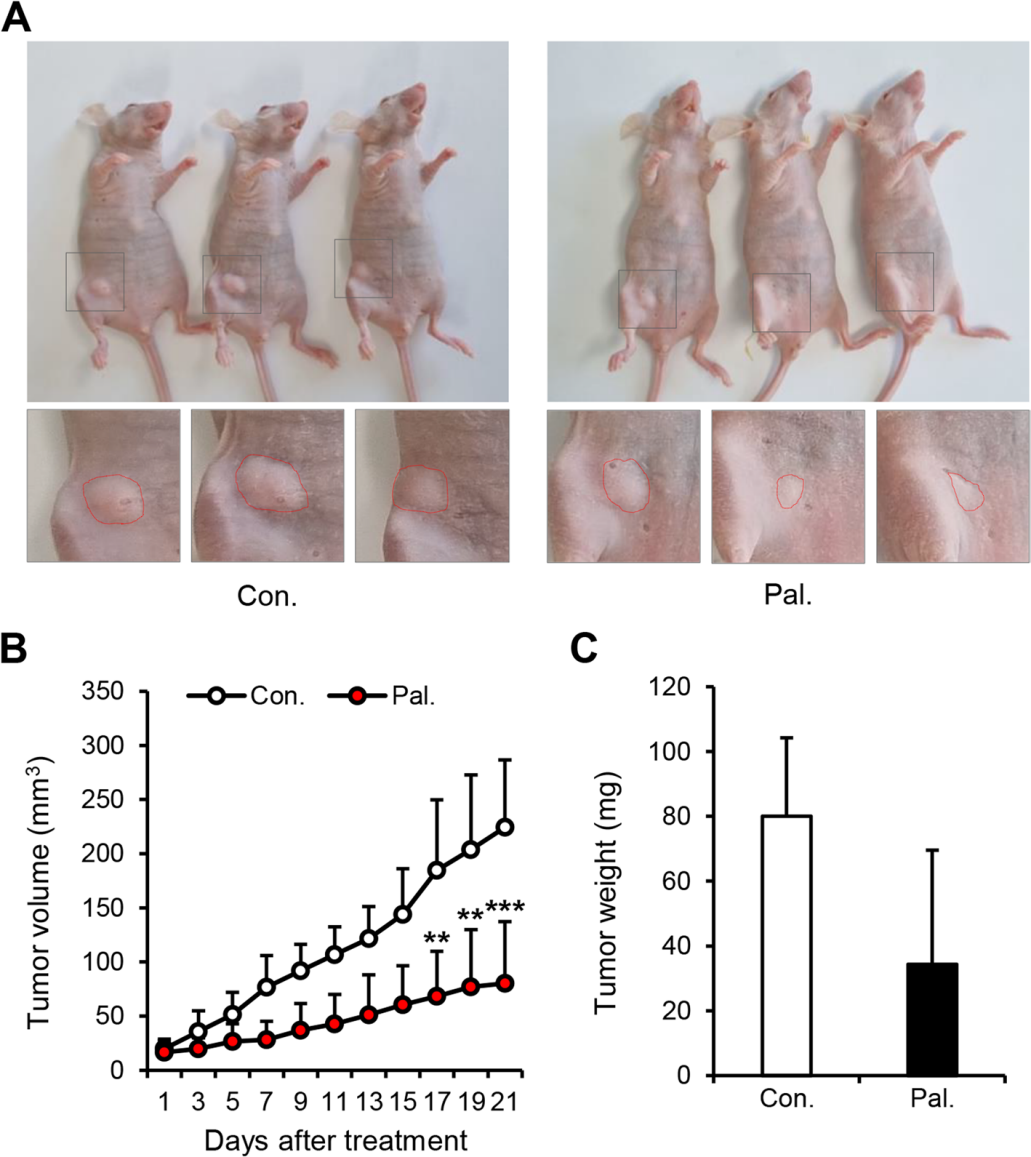
Palmatine suppresses tumor progression in an in vivo CMT-U27 mouse xenograft model. **A** Tumor development at 21 days after intraperitoneal injection of Palmatine (50 mg/kg) and DMSO in PBS (control) in BALB/c nude mice. The dotted lines in the magnified images demarcate CMT masses. **B** Comparison of tumor volume in treatment group and control group over 21 days. **C** Comparison of the weight of tumors harvested from treatment and control group after 21 days of injection. Unless otherwise denoted: *n* = 5 each group. Values are mean ± SD. ** *p* < 0.01; *** *p* < 0.001 versus control. Con., control; Pal., palmatine

To confirm the tumor growth-inhibitory effects of palmatine, we used an in vivo nude mouse xenograft model. After palmatine treatment for 21 days, the tumors in the treatment group were smaller than those in the control group (Fig. 1A). In addition, the volume difference between the treatment and control groups gradually increased over the 21 days of injection, reaching significance (*p* < 0.01) at day 17. The tumor volume in the control group was 184.63 mm^3^ and that of the treatment group was 68.15 mm^3^. Moreover, on the 21^st^ day, the tumor volume in the control group was 2.8 times larger (*p* < 0.001) than that in the treatment group (Fig. 1B). However, there was a difference in the average value (80 mg in the control group and 34.3 mg in the treatment group), though no significant difference appeared on the graph (Fig. 1C). These results show that palmatine inhibited tumor growth in an in vivo nude mouse model.

### Palmatine induces cell death and inhibits cell proliferation in CMT-U27

**Fig. 2:**
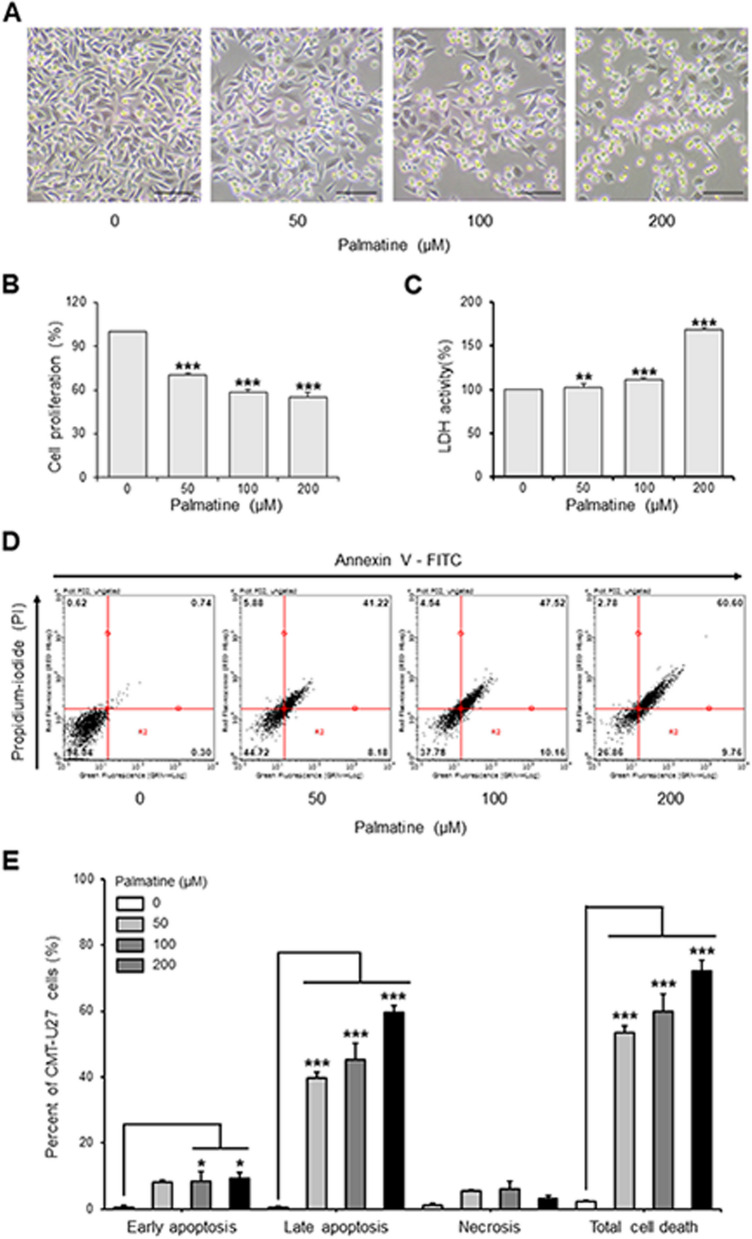
Palmatine induces cell death of CMT-U27 cells. **A** Images showing morphological changes on CMT-U27 cells after palmatine-treatment. Magnification, 100 × . Scale bar, 50 µm. **B**, **C** Dose-dependent comparison of CMT-U27 cell proliferation and LDH activity. Values are mean ± SD. ** *p* < 0.01; *** *p* < 0.001 versus untreated cells by one-way ANOVA followed by Bonferroni post-hoc test. **D** Apoptosis in CMT-U27 cells was measured by flow cytometry with Annexin V-FITC staining. **E** Percentage of CMT-U27 cells in early, late apoptosis, necrosis, and total cell death when treated with palmatine by concentration. Values are mean ± SD. * *p* < 0.05; *** *p* < 0.001 versus untreated cells by one-way ANOVA followed by Bonferroni post-hoc test

Morphological observations and MTS assays were performed to confirm whether palmatine suppresses cell proliferation of CMT-U27. Morphological images showed that CMT-U27 cells became round in a dose-dependent manner (Fig. 2A). Palmatine greatly suppressed (*p* < 0.001) cell proliferation in all treatment groups, and it was 70%, 58%, and 55% less compared to the control group at 50 μM, 100 μM, and 200 μM, respectively (Fig. 2B). In addition, lactate dehydrogenase (LDH) and annexin V-PI assays were performed to determine the effects of palmatine on cell death in CMT-U27 cells. The LDH assay showed an increase of 2.1% at 50 μM (*p* < 0.01), 11.5% at 100 μM (*p* < 0.001), and 68.4% at 200 μM (*p* < 0.001) compared with the control group, with a noticeable difference at 100 μM (Fig. 2C). In addition, annexin V-PI assay confirmed that palmatine treatment increased total cell death in a dose-dependent manner compared to control group (Fig. 2D). When percentage, cells in early apoptosis were about 0.55%, 8.2%, 8.4%, and 9.16% for 0 μM, 50 μM, 100 μM (*p* < 0.05) and 200 μM (*p* < 0.05), respectively, and cells in late apoptosis were about 0.66%, 39.6% (*p* < 0.001), 45.3% (*p* < 0.001), and 59.7% (*p* < 0.001), respectively. Cells in necrosis were about 0.99%, 5.6%, 6.1%, and 3.3% for 0 μM, 50 μM, 100 μM and 200 μM, respectively. Collectively, the total cell death was approximately 2.2%, 53,4%, 59.8%, and 72.2%, respectively. Among them, the cells belonging to late apoptosis and total cell death increased dose-dependent manner with highly significant differences (Fig. 2E). These results indicate that palmatine suppresses cell proliferation and induces cell death in CMT-U27 cells.

### Palmatine exerts anti-cancer effects on CMT-U27 by inhibiting PI3K/AKT pathway

**Fig. 3:**
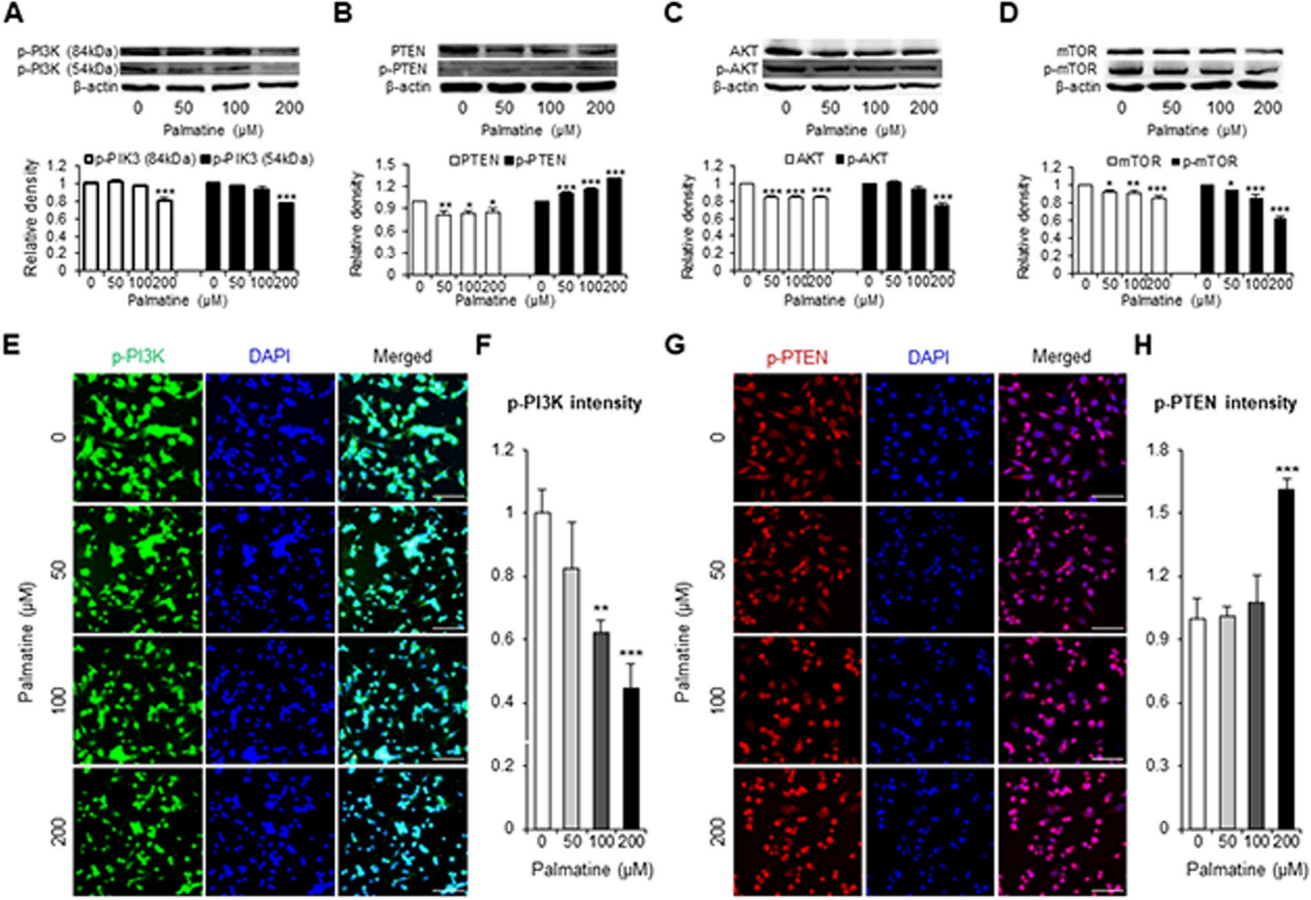
Palmatine regulates the PI3K/AKT signaling pathway in CMT-U27 cells. **A-D** CMT-U27 cells grown in 6-well plates and then cultured in fresh media containing various concentrations of palmatine for 18 h at 37ºC. After treatment, the cells were harvested for western blotting to quantify the expression of p-PI3K (**A**), PTEN, p-PTEN (**B**), AKT, p-AKT (**C**), mTOR, p-mTOR (**D**), and β-actin. **E–H** CMT-U27 cells grown in 24-well plates and then cultured in fresh media containing various concentrations of palmatine for 18 h at 37ºC. After treatment, immunocytochemistry was performed to quantify the expression of p-PI3K (**E**) and p-PTEN (**G**). Scale bar, 50 µm. Values are mean ± SD. * *p* < 0.05, ** *p* < 0.01, *** *p* < 0.001 versus untreated cells by one-way ANOVA followed by Bonferroni post-hoc test

**Fig. 4:**
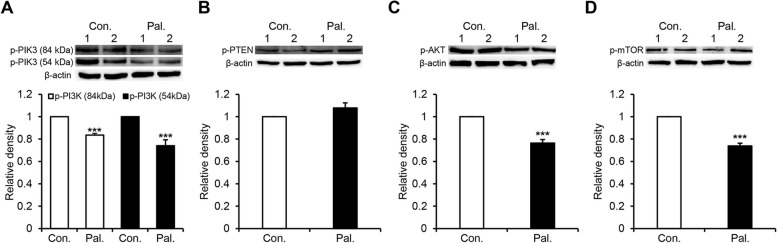
Palmatine inhibits PI3K/AKT pathway and increases PTEN activity in an in vivo CMT-U27 xenograft models. Tumors of CMT-U27 xenograft model injected with palmatine (treatment group) or DMSO in PBS (control group) for 21 days were collected and western blotting was performed. **A-D** Images and quantification of the protein expression of p-PI3K (**A**), p-PTEN (**B**), p-AKT (**C**), p-mTOR (**D**) and β-actin in tumors. Values are mean ± SD. *** p < 0.001 versus control by unpaired Student’s *t*-test. Con., control; Pal., palmatine

To confirm how palmatine inhibits cell proliferation and induces cell death, the PI3K/AKT pathway was identified by western blotting and immunocytochemistry in vitro. Western blot analysis showed that the protein expression levels of PI3K, AKT, and mTOR and the components of the PI3K/AKT pathway, except for p-PTEN, decreased in a dose-dependent manner. Particularly, at 200 μM, the expression of p-PI3K (84 kDa) and p-PI3K (54 kDa) was sharply reduced (*p* < 0.001) by about 20% and 22%, respectively, compared with the control group (Fig. 3A). AKT expression was remarkably reduced (*p* < 0.001) by approximately 16% in all treatment groups compared with the control group. Furthermore, p-AKT expression was significantly reduced by approximately 25% at 200 μM (*p* < 0.001) compared with that in the control group (Fig. 3C). The expression of mTOR was reduced by approximately 8% at 50 μM (*p* < 0.05), 10% at 100 μM (*p* < 0.01) and 16% at 200 μM (*p* < 0.001) compared with the control group. And the expression of p-mTOR was notably decreased (*p* < 0.001) by 15% and 39% at 100 μM and 200 μM, respectively, compared with the control group (Fig. 3D). Similar to the downregulation of these proteins, PTEN expression decreased by approximately 20% at 50 μM (*p* < 0.01), 17% at 100 μM (*p* < 0.05), and 16% at 200 μM (*p* < 0.05) compared with the control group. In contrast, the expression of p-PTEN was increased (*p* < 0.001) in a dose-dependent manner by 10%, 18%, and 31%, respectively, at 50 μM, 100 μM, and 200 μM compared with the control group (Fig. 3B). Subsequently, we performed immunocytochemistry to confirm the western blotting results for PI3K and p-PTEN. p-PI3K expression decreased by 38% and 55% at 100 μM (*p* < 0.01) and 200 μM (*p* < 0.001), respectively, compared with the control group, confirming a significant decrease, as shown in the western blot results (Fig. 3E, F). In addition, p-PTEN expression significantly increased (*p* < 0.001) by 60% at 200 μM compared with the control group, and a significant increase was observed as shown in the western blot (Fig. 3G, H). To confirm whether the inhibitory action of palmatine on the PI3K/AKT pathway occurs equally in vitro and in vivo, western blotting was performed using tumor tissues from CMT-U27 nude mouse xenograft models. Western blotting analysis showed that the in vitro and in vivo results were similar. Specifically, p-PI3K expression in the treatment group was noticeably reduced by 17% and 26% at 84 kDa (*p* < 0.001) and 54 kDa (*p* < 0.001), respectively, compared with that in the control group (Fig. 4A). p-AKT expression was significantly decreased (*p* < 0.001) by 25% compared with that in the control group (Fig. 4C). Similarly, the expression of p-mTOR was reduced by approximately 26% in the treatment group compared with that in the control group (*p* < 0.001) (Fig. 4D). The expression of p-PTEN increased by approximately 7% in the treatment group compared with that in the control group, but the difference was not significant (Fig. 4B). These results showed that palmatine inhibits the PI3K/AKT pathway both in vivo and in vitro.

### Palmatine inhibits angiogenesis of canine mammary gland tumors

**Fig. 5:**
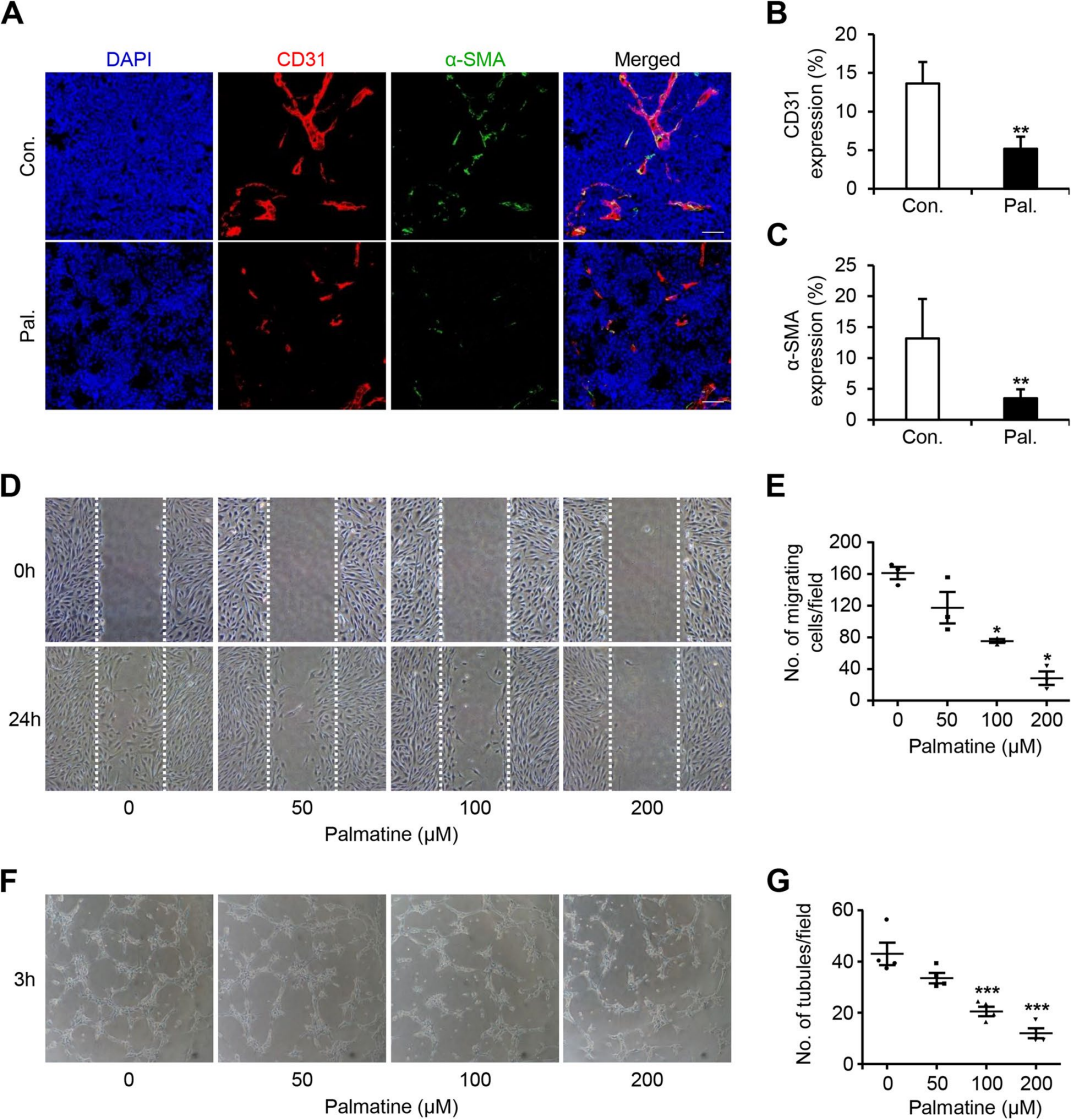
Palmatine suppresses angiogenesis in CMTs and CnAoECs. **A-C** Fluorescent images and quantification of CD31 and α-SMA expression differences between treatment and control groups. Scale bar, 50 µm. Values are mean ± SD. ** *p* < 0.01 versus control by unpaired Student’s *t*-test. Con., control; Pal., palmatine. **D**, **E** CnAOECs grown in 6-well plates and scratched by 1000 μl tip. Then, CnAOECs cultured in fresh media containing various concentrations of palmatine for 24 h at 37ºC. Images showing CnAOECs migration after palmatine treatment in a dose-dependent manner and quantification of the number of migrated cells. Values are mean ± SD. * *p* < 0.05 versus untreated cells by one-way ANOVA followed by Bonferroni post-hoc test. **F**, **G** CnAOECs were seeded in matrigel-coated 6-well plates and incubated for 3 h at 37ºC. Images showing CnAOECs tube formation after palmatine treatment in a dose-dependent manner and quantification of the number of tubules. Values are mean ± SD. *** *p* < 0.001 versus untreated cells by one-way ANOVA followed by Bonferroni post-hoc test

To confirm whether palmatine inhibited angiogenesis in CMTs, immunohistochemistry was performed using tumors from in vivo CMT-U27 nude mouse xenograft models. Confocal microscopy revealed that more blood vessels were formed in the tumors of the control group than in those of the treatment group (Fig. 5A). Similarly, the expression of CD31 and α-SMA, which are vascular factors, was significantly decreased (*p* < 0.01) in the treatment group. CD31 was expressed by approximately 14% and 5% in the control and treatment groups, respectively, and α-SMA was expressed by approximately 13% and 3.5% in the control and treatment groups, respectively (Fig. 5B, C). We also performed migration and tube formation assays using CnAOECs to determine whether palmatine affected angiogenesis and metastasis in canines. The migration assay results showed that the numbers of migrated cells were 161, 117, 75, and 29 at 0 μM, 50 μM, 100 μM (*p* < 0.05), and 200 μM (*p* < 0.05), respectively. Thus, CnAOECs migration decreased in a dose-dependent manner (Fig. 5D, E). In the tube formation assay, it was observed that 43, 34, 21, and 12 tubes were formed at 0 μM, 50 μM, 100 μM (*p* < 0.001), and 200 μM (*p* < 0.001), respectively, which decreased in a dose-dependent manner similar to migration (Fig. 5F, G). Collectively, palmatine appears to have an anti-angiogenic function, because it inhibits the migration and tube formation of CnAOECs and angiogenesis in in vivo CMT-U27 nude mouse xenograft models.

### Palmatine inhibits the metastasis of canine mammary gland tumor cells

**Fig. 6:**
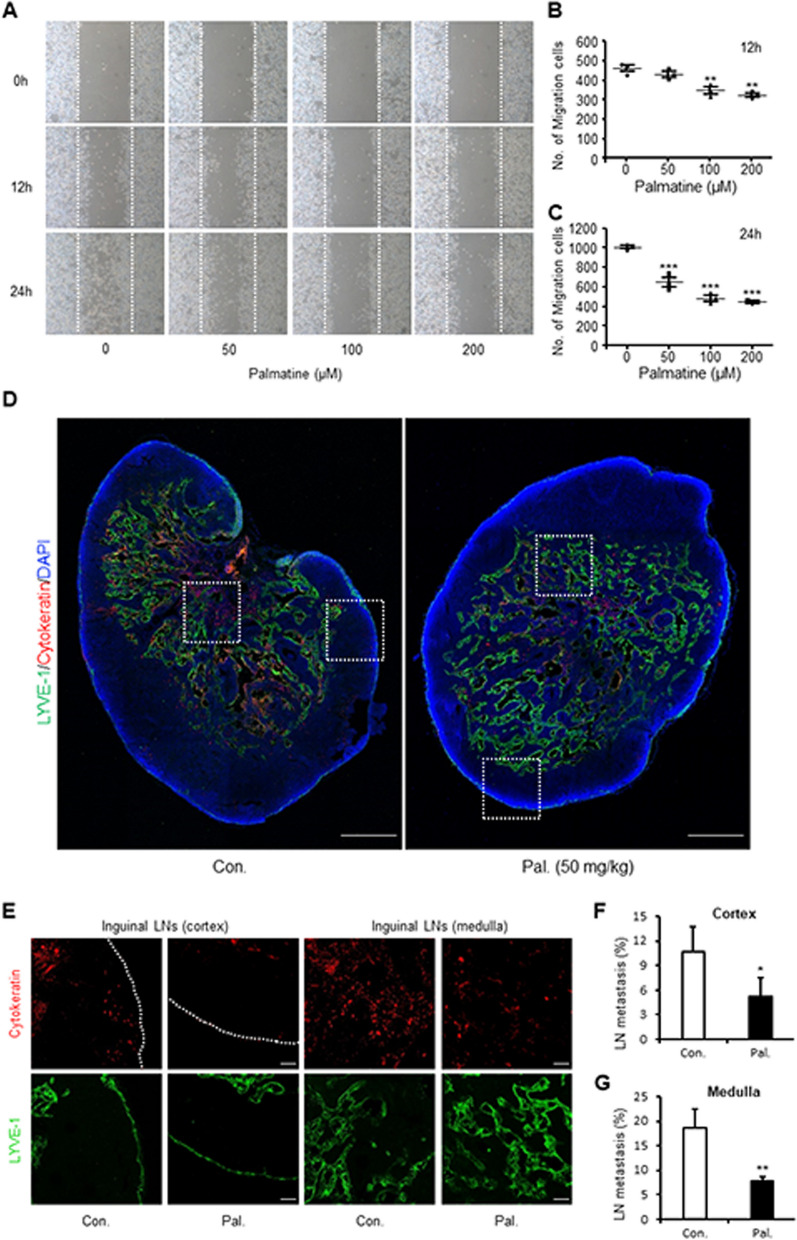
Palmatine restrains the migration and metastasis of CMT cells. **A-C** CMT-U27 cells grown in 6-well plates and scratched by 1000 μl tip. Then, CMT-U27 cells cultured in fresh media containing various concentrations of palmatine for 24 h at 37ºC. Images showing CMT-U27 cells migration after palmatine treatment in a dose- and time-dependent manner and quantification of the number of migrated cells over time. Magnification, 40 × . Values are mean ± SD. ** *p* < 0.01, *** *p* < 0.001 versus untreated cells by one-way ANOVA followed by Bonferroni post-hoc test. **D** Fluorescent images showing the lymph nodes of treatment and control groups stained for cytokeratin and LYVE-1. Scale bar, 400 μm. **E–G** Magnified images and quantification of lymph node metastases in the cortex and medulla regions in the control and treatment groups. The dotted lines demarcate the margin of the lymph node. Scale bar, 50 μm. Values are mean ± SD. * *p* < 0.05, ** *p* < 0.01 versus control by one-way ANOVA followed by Bonferroni post-hoc test. Con., control; Pal., palmatine

A migration assay was performed to confirm whether palmatine inhibits the migration of CMT-U27 cells in vitro. Microscopic observation revealed that migration decreased in a concentration-dependent manner at both 12 and 24 h (Fig. 6A). When we quantified this, approximately 460 cells at 0 μM, 430 cells at 50 μM, 348 cells at 100 μM (*p* < 0.01), and 321 cells at 200 μM (*p* < 0.01) migrated at 12 h after palmatine treatment (Fig. 6B). After 24 h of treatment, approximately 1007 cells migrated at 0 μM, 651 cells at 50 μM (*p* < 0.001), 484 cells at 100 μM (*p* < 0.001), and 445 cells at 200 μM (*p* < 0.001) (Fig. 6C). In summary, palmatine significantly inhibited migration at 100 μM after 12 h of treatment and effectively inhibited migration at 50 μM after 24 h. Next, immunohistochemistry was performed on the inguinal lymph nodes adjacent to the tumor to confirm whether migration to other tissues was inhibited in the in vivo CMT-U27 xenograft model. Under the microscope, more cytokeratin, a tumor factor, was observed in the lymph nodes of the control group than in those of the treatment group (Fig. 6D). In addition, in the treatment group, most cytokeratin was present around the lymphatic vessels, whereas in the control group, it was present not only around the lymphatic vessels but also in the cortex of the lymph nodes (Fig. 6E). When we evaluated the cytokeratin expression in the control and treatment groups, a significant difference was found in both the medulla (*p* < 0.01) and cortex (*p* < 0.05), as the control group had more than twice as many metastases than the treatment group (Fig. 6F, G). Based on these results, palmatine is considered to inhibit the metastasis of canine mammary gland tumor cells.

## Discussion

Palmatine is a natural isoquinoline alkaloid found in traditional Chinese medicines, such as *Tinospora cordifolia, Corydalis yanhusuo, Phellodendron amurense, Tinospora sagittate,* and *Stephania yunnanensis* [28–30, 41, 42]. Previous studies have revealed that palmatine has several therapeutic properties including neuroprotective, anti-cancer, antibacterial, and anti-inflammatory activities [43–46]. In the anti-cancer field, which is the focus of our study, palmatine has been reported to have anti-cancer effects on various cancer cells [35, 36]. However, no such studies have been conducted on canine cancer. Furthermore, the effects of palmatine have not been verified in vivo. Therefore, this study aimed to investigate the anti-cancer effects and the underlying mechanisms of action of palmatine in canine cancers on in vitro and in vivo. Our results demonstrated that palmatine inhibits the PI3K/AKT pathway, leading to an anti-cancer effect in CMTs.

Our findings indicated that palmatine inhibited tumor growth in CMT-U27 xenograft nude mouse models, reducing their volume and weight in vivo (Fig. 1). In addition, palmatine induced cell death and inhibited the proliferation of CMT-U27 cells in vitro (Fig. 2). Annexin-V / PI staining results showed that cells in early and late apoptosis increased by a significant difference, while necrotic cells did not. The total number of cell death also increased by a significant difference, confirming that palmatine induced apoptosis in CMT-U27, resulting in a decrease in the number of cells. These results are consistent with those of previous studies and were further validated in additional experiments. Based on these findings, we propose that palmatine exerts similar effects on CMT-U27 cells as on human cancer cells.

Next, we determined the mechanism by which palmatine exerts its anti-cancer effects. Previous studies have shown that palmatine inhibits the mTORC1 pathway in human prostate cancer cells. However, it is unclear whether this occurs via the PI3K/AKT pathway [47]. Hence, in this study, we explored the effect of palmatine on the PI3K/AKT signaling pathway, which is located upstream of mTOR signaling, and on the mTOR pathway. We also measured the expression of PTEN, the primary negative regulator of the PI3K/AKT pathway. The PI3K/AKT pathway is involved in tumor metabolism including proliferation, cell cycle, and apoptosis [48]. Consequently, PI3K/AKT pathway inhibitors have been used as therapeutic agents to impede tumor growth [16]. Our results showed that palmatine decreased the expression of PI3K, AKT, and mTOR in CMT-U27 cells (Fig. 3A, B, and D). Furthermore, in vivo experiments confirmed that the protein expression in the tumor tissue of the treatment group was lower than that in the tumor tissue of the control group (Fig. 4A, C, and D). In addition, in in vitro experiments, the expression of PTEN decreased, while that of p-PTEN increased (Fig. 3B). In in vivo experiments, p-PTEN levels also increased (Fig. 4B). Interestingly, our results showed that palmatine decreased the expression of PTEN and increased the expression of p-PTEN, although PTEN is a widely known negative regulator of PI3K. However, in accordance with previous studies, it determined that the results of decreased PTEN expression and increased p-PTEN expression, along with decreased p-PI3K expression were not contradictory. Mukherjee R et al., showed that PTEN expression was reduced when treated with a PI3K inhibitor. Additionally, it demonstrated that PTEN expression is regulated by mTOR-dependent translation [49]. Another study identified the upregulation of both p-PTEN expression and PTEN gene when treated with PI3K or AKT inhibitors and demonstrated that p53 is involved in the regulation of PTEN expression [50]. Consistent with, our results might be a possible consequence of treatment with palmatine, as a PI3K inhibitor. However, the exact mechanism of PTEN regulation will need to be studied in the future.

**Fig. 7:**
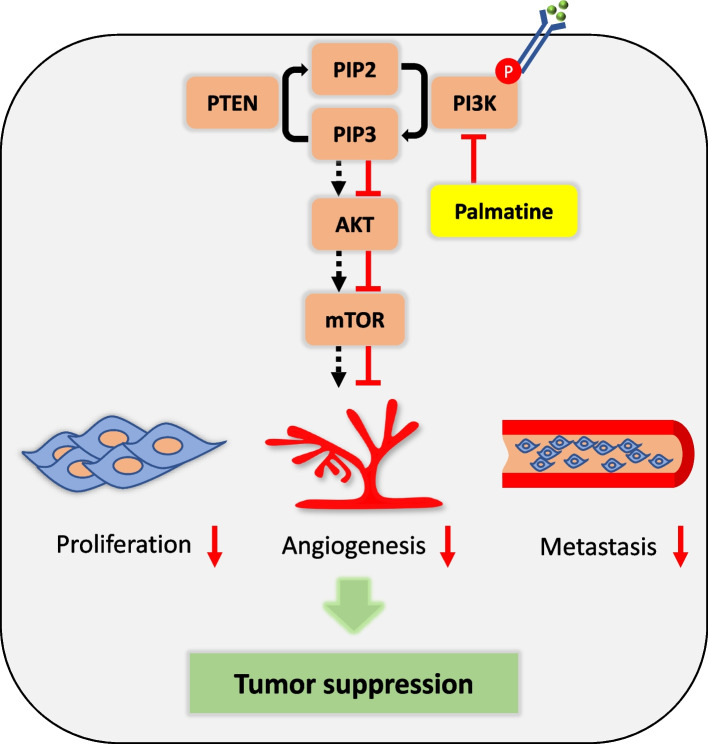
Schematic diagram of the mechanism of anti-cancer effect of palmatine

Tumors require a sufficient supply of oxygen and nutrients for proliferation and survival. Although the host can initially provide some supply, it quickly becomes insufficient to meet the demands of the tumor. Thus, tumors develop their own blood vessel networks through a process known as angiogenesis [51]. Angiogenesis is necessary for tumor growth and metastasis [52]. The knockout of angiogenesis suppressors has been shown to increase tumor angiogenesis and metastasis [53, 54]. Moreover, angiogenesis and metastasis are reduced by the application of PI3K/AKT pathway inhibitors, demonstrating that this pathway regulates them [55–57]. While a previous study demonstrated that the alkaloids noscapine and sinomenine have anti-angiogenic effects [58, 59], the effect of palmatine on angiogenesis has yet to be studied. In this study, we confirmed the effect of palmatine on angiogenesis and metastasis by reducing the expression of the PI3K/AKT pathway in in vivo CMT-U27 xenograft tumor tissue and CnAOECs in vitro. In the in vivo experiment, we found that the expression of CD31 and α-SMA, which are vascular markers, was decreased in the palmatine-treated group. Furthermore, in vitro, palmatine inhibited CnAOECs migration and tube formation in a dose-dependent manner. The inhibition of angiogenesis by palmatine was consistent with previous studies on the anti-angiogenic effects of PI3K/AKT pathway inhibitors [60, 61]. In addition, the inhibitory effect of palmatine on CnAOECs indicated that palmatine might also have an inhibitory effect on canine angiogenesis. Next, we measured the expression of cytokeratin, a tumor factor, in the inguinal lymph nodes adjacent to the tumor in an in vivo CMT-U27 xenograft model to evaluate the degree of metastasis. These results showed that cytokeratin was expressed at lower levels in the lymph nodes of the treatment group than in those of the control group, indicating that the treatment group had fewer metastases than the control group. Consistent with studies that demonstrate that palmatine is an inhibitor of the PI3K/AKT pathway [57, 62], our findings also indicate that palmatine inhibits proliferation, angiogenesis and metastasis by suppressing the PI3K/AKT signaling pathway in tumor tissues (Fig. 7).

A limitation of our paper is that we only confirmed the efficacy of palmatine against CMT-U27 among canine mammary gland tumor cell lines. Therefore, further studies on its efficacy in other cell lines are needed.

## Conclusion

In conclusion, this study investigated the anti-cancer effects of palmatine on canine tumor cells and its mechanism of action. Our results demonstrated that palmatine has anti-cancer effects on CMT-U27 cells, including the induction of cell death and inhibition of angiogenesis and metastasis, which are exerted through inhibition of the PI3K/AKT pathway. Furthermore, the effects of palmatine are similar to those of PI3K/AKT pathway inhibitors in humans. Therefore, we suggest that palmatine can be used as a PI3K/AKT pathway inhibitor to treat CMTs.

### Supplementary Information

**Additional file 1:** **Figure S1.** Each figure (A, B, C and D) represents the uncropped scan of the western blots shown in Fig. 3, respectively. Band images shown in Fig. 3 are marked in red box. **Figure S2.** Each figure (A, B, C and D) represents the uncropped scan of the western blots shown in Fig. 4, respectively. Band images shown in Fig. 4 are marked in red box. The p-PTEN in the (B) gel is lightly banded, so we used an image with increased contrast in the manuscript.

## Data Availability

All data generated or analysed during this study are included in this published article.

## References

[1] Dorn CR, Taylor DO, Schneider R, Hibbard HH, Klauber MR (1968). Survey of animal neoplasms in Alameda and contra costa counties, California. II. Cancer morbidity in dogs and cats from Alameda County. J Natl Cancer Inst.

[2] Withrow S, Vail D, Page R (2001). Small animal oncology.

[3] Gamlem H, Nordstoga K, Glattre E (2008). Canine neoplasia – Introductory paper. APMIS.

[4] Hampe JF, Misdorp W (1974). Tumours and dysplasias of the mammary gland. Bull World Health Organ.

[5] Straw R. Treatment of mammary gland tumors and perianal neoplasia. pp. 672-675. In: The North American Veterinary Conference (NAVC) Orlando, Florida, 2005. 2005.

[6] Misdorp W. Tumors of the Mammary Gland. In: Tumors in Domestic Animals. Ames: Blackwell Publishing Professional; 2002. p. 575–606.

[7] Karayannopoulou M, Kaldrymidou E, Constantinidis TC, Dessiris A (2001). Adjuvant Post-operative Chemotherapy in Bitches with Mammary Cancer. J Vet Med Ser A.

[8] Simon D, Schoenrock D, Baumgärtner W, Nolte I (2006). Postoperative adjuvant treatment of invasive malignant mammary gland tumors in dogs with doxorubicin and docetaxel. J Vet Intern Med.

[9] Marconato L, Lorenzo RM, Abramo F, Ratto A, Zini E (2008). Adjuvant gemcitabine after surgical removal of aggressive malignant mammary tumours in dogs. Veterinary and Comparative Oncology.

[10] Sorenmo KU, Worley DR, Zappulli V, Vail DM, Thamm DH, Liptak JM (2019). 28 - Tumors of the Mammary Gland. Withrow and MacEwen's Small Animal Clinical Oncology.

[11] Stratmann N, Failing K, Richter A, WEHREND A,  (2008). Mammary tumor recurrence in bitches after regional mastectomy. Vet Surg.

[12] Chang SC, Chang CC, Chang TJ, Wong ML (2005). Prognostic factors associated with survival two years after surgery in dogs with malignant mammary tumors: 79 cases (1998–2002). J Am Vet Med Assoc.

[13] Engelman JA, Luo J, Cantley LC (2006). The evolution of phosphatidylinositol 3-kinases as regulators of growth and metabolism. Nat Rev Genet.

[14] Graupera M, Potente M (2013). Regulation of angiogenesis by PI3K signaling networks. Exp Cell Res.

[15] Carrera AC, Anderson R (2019). The cell biology behind the oncogenic PIP3 lipids. J Cell Sci.

[16] Castel P, Toska E, Engelman JA, Scaltriti M (2021). The present and future of PI3K inhibitors for cancer therapy. Nat Cancer.

[17] Mei ZB, Duan CY, Li CB, Cui L, Ogino S (2016). Prognostic role of tumor PIK3CA mutation in colorectal cancer: a systematic review and meta-analysis. Ann Oncol.

[18] Herberts C, Murtha AJ, Fu S, Wang G, Schönlau E, Xue H, Lin D, Gleave A, Yip S, Angeles A (2020). Activating AKT1 and PIK3CA mutations in metastatic castration-resistant prostate cancer. Eur Urol.

[19] Iranpour M, Nourian M, Saffari S, Samizadeh E, Mirghafori M, Iravani S, Ghafouri-Fard S (2019). PIK3CA mutation analysis in Iranian patients with gastric cancer. Iran Biomed J.

[20] Martínez-Sáez O, Chic N, Pascual T, Adamo B, Vidal M, González-Farré B, Sanfeliu E, Schettini F, Conte B, Brasó-Maristany F (2020). Frequency and spectrum of PIK3CA somatic mutations in breast cancer. Breast Cancer Res.

[21] MacEwen EG, Patnaik AK, Harvey HJ, Panko WB (1982). Estrogen receptors in canine mammary tumors. Cancer Res.

[22] Block GE, Jensen EV, Polley TZ (1975). The prediction of hormonal dependency of mammary cancer. Ann Surg.

[23] Chu LL, Rutteman GR, Kong JM, Ghahremani M, Schmeing M, Misdorp W, van Garderen E, Pelletier J (1998). Genomic organization of the canine p53 gene and its mutational status in canine mammary neoplasia. Breast Cancer Res Treat.

[24] Queiroga FL, Raposo T, Carvalho MI, Prada J, Pires I (2011). Canine mammary tumours as a model to study human breast cancer: most recent findings. In Vivo.

[25] Lee KH, Hwang HJ, Noh HJ, Shin TJ, Cho JY (2019). Somatic mutation of PIK3CA (H1047R) is a common driver mutation hotspot in canine mammary tumors as well as human breast cancers. Cancers (Basel).

[26] Burrai GP, Tanca A, De Miglio MR, Abbondio M, Pisanu S, Polinas M, Pirino S, Mohammed SI, Uzzau S, Addis MF (2015). Investigation of HER2 expression in canine mammary tumors by antibody-based, transcriptomic and mass spectrometry analysis: is the dog a suitable animal model for human breast cancer?. Tumor Biology.

[27] Kim JH, Im KS, Kim NH, Yhee JY, Nho WG, Sur JH (2011). Expression of HER-2 and nuclear localization of HER-3 protein in canine mammary tumors: Histopathological and immunohistochemical study. Vet J.

[28] Kumar P, Srivastava V, Chaturvedi R, Sundar D, Bisaria VS (2017). Elicitor enhanced production of protoberberine alkaloids from in vitro cell suspension cultures of Tinospora cordifolia (Willd.) Miers ex Hook. F. & Thoms. Plant Cell Tissue Organ Culture (PCTOC).

[29] Zhang Q, Chen C, Wang F-Q, Li C-H, Zhang Q-H, Hu Y-J, Xia Z-N, Yang F-Q (2016). Simultaneous screening and analysis of antiplatelet aggregation active alkaloids from Rhizoma Corydalis. Pharm Biol.

[30] Sun M, Xu L, Peng Y, Liu T, Zhang Y, Zhou Z (2016). Multiscale analysis of the contents of palmatine in the Nature populations of Phellodendron amurense in Northeast China. Journal of Forestry Research.

[31] Zhao Y, Ma J, Gao Y, Song C, Ma J, Zhang J, Wang Z. Pharmacopoeia of the People’s Republic of China 2015. 10th ed. London: The Stationery Office/Tso; 2017.

[32] Ou J, Hsieh W, Lin I, Chang Y, Chen I (2003). The catalogue of medicinal plant resources in Taiwan.

[33] Meng F-C, Wu Z-F, Yin Z-Q, Lin L-G, Wang R, Zhang Q-W (2018). Coptidis rhizoma and its main bioactive components: recent advances in chemical investigation, quality evaluation and pharmacological activity. Chin Med.

[34] Ríos JL, Francini F, Schinella GR (2015). Natural products for the treatment of type 2 diabetes mellitus. Planta Med.

[35] Zhang L, Li J, Ma F, Yao S, Li N, Wang J, Wang Y, Wang X, Yao Q (2012). Synthesis and cytotoxicity evaluation of 13-n-alkyl berberine and palmatine analogues as anticancer agents. Molecules.

[36] Costa EV, Cruz PEOd, Pinheiro MLB, Marques FA, Ruiz ALT, Marchetti GM, Carvalho JEd, Barison A, Maia BHL (2013). Aporphine and tetrahydroprotoberberine alkaloids from the leaves of Guatteria friesiana (Annonaceae) and their cytotoxic activities. J Braz Chem Soc.

[37] Grabarska A, Wróblewska-Łuczka P, Kukula-Koch W, Łuszczki JJ, Kalpoutzakis E, Adamczuk G, Skaltsounis AL, Stepulak A (2021). Palmatine, a bioactive protoberberine alkaloid isolated from Berberis cretica, inhibits the growth of human estrogen receptor-positive breast cancer cells and acts synergistically and additively with Doxorubicin. Molecules.

[38] Ativui S, Danquah CA, Ossei PPS, Ofori M (2022). Palmatine attenuates metastatic lung colonization of triple negative breast cancer cells. Front Pharmacol.

[39] Zheng Q, Pei Y, Li Y, Chen H, Zhao Z, Hu Q. Palmatine suppresses acute myeloid leukemia (AML) by inducing pyroptosis via ROS/PI3K/AKT signaling. In: Research Square. 2022.

[40] Torrance CJ, Agrawal V, Vogelstein B, Kinzler KW (2001). Use of isogenic human cancer cells for high-throughput screening and drug discovery. Nat Biotechnol.

[41] Rong J, Wei H, Huang H, Wu H, Xu M, Li Y. Determination of Organochlorine and Pyrethroid Pesticides in Tea by Gas Chromatography-Mass Spectrometry Using Hydroxylated Multi-walled Carbon Nanotubes as Dispersive Solid-phase Extraction Sorbent. J Instrumental Anal. 2016;35(1):8–15.

[42] Xin A, Zhang Y, Zhang Y, Di D, Liu J (2018). Development of an HPLC-DAD method for the determination of five alkaloids in Stephania yunnanensis Lo and in rat plasma after oral dose of Stephania yunnanensis Lo extracts. Biomed Chromatogr.

[43] Long J, Song J, Zhong L, Liao Y, Liu L, Li X (2019). Palmatine: a review of its pharmacology, toxicity and pharmacokinetics. Biochimie.

[44] Dhingra D, Bhankher A (2014). Behavioral and biochemical evidences for antidepressant-like activity of palmatine in mice subjected to chronic unpredictable mild stress. Pharmacol Rep.

[45] Deng Y, Zhang M, Luo H (2012). Identification and antimicrobial activity of two alkaloids from traditional Chinese medicinal plant Tinospora capillipes. Ind Crops Prod.

[46] Chen G, Xu Y, Jing J, Mackie B, Zheng X, Zhang X, Wang J, Li X (2017). The anti-sepsis activity of the components of Huanglian Jiedu Decoction with high lipid A-binding affinity. Int Immunopharmacol.

[47] Hambright HG, Batth IS, Xie J, Ghosh R, Kumar AP (2015). Palmatine inhibits growth and invasion in prostate cancer cell: potential role for rpS6/NFκB/FLIP. Mol Carcinog.

[48] Barra F, Evangelisti G, Ferro Desideri L, Di Domenico S, Ferraioli D, Vellone VG, De Cian F, Ferrero S (2019). Investigational PI3K/AKT/mTOR inhibitors in development for endometrial cancer. Expert Opin Investig Drugs.

[49] Mukherjee R, Vanaja KG, Boyer JA, Gadal S, Solomon H, Chandarlapaty S, Levchenko A, Rosen N (2021). Regulation of PTEN translation by PI3K signaling maintains pathway homeostasis. Mol Cell.

[50] Naderali E, Valipour B, Khaki AA, Soleymani Rad J, Alihemmati A, Rahmati M, Nozad Charoudeh H (2019). Positive effects of PI3K/Akt signaling inhibition on PTEN and P53 in prevention of acute lymphoblastic leukemia tumor cells. Adv Pharm Bull.

[51] Folkman J (1986). How is blood vessel growth regulated in normal and neoplastic tissue? G.H.A Clowes memorial award lecture. Cancer Res.

[52] Folkman J (1971). Tumor angiogenesis: therapeutic implications. N Engl J Med.

[53] Lee YJ, Koch M, Karl D, Torres-Collado AX, Fernando NT, Rothrock C, Kuruppu D, Ryeom S, Iruela-Arispe ML, Yoon SS (2010). Variable inhibition of thrombospondin 1 against liver and lung metastases through differential activation of metalloproteinase ADAMTS1. Cancer Res.

[54] Hawighorst T, Velasco P, Streit M, Hong Y-K, Kyriakides TR, Brown LF, Bornstein P, Detmar M (2001). Thrombospondin-2 plays a protective role in multistep carcinogenesis: a novel host anti-tumor defense mechanism. EMBO J.

[55] Garlich JR, De P, Dey N, Su JD, Peng X, Miller A, Murali R, Lu Y, Mills GB, Kundra V (2008). A vascular targeted pan phosphoinositide 3-kinase inhibitor prodrug, SF1126, with antitumor and antiangiogenic activity. Can Res.

[56] Maira SM, Stauffer F, Brueggen J, Furet P, Schnell C, Fritsch C, Brachmann S, Chène P, De Pover A, Schoemaker K (2008). Identification and characterization of NVP-BEZ235, a new orally available dual phosphatidylinositol 3-kinase/mammalian target of rapamycin inhibitor with potent in vivo antitumor activity. Mol Cancer Ther.

[57] Li B, Xu WW, Lam AKY, Wang Y, Hu HF, Guan XY, Qin YR, Saremi N, Tsao SW, He QY (2017). Significance of PI3K/AKT signaling pathway in metastasis of esophageal squamous cell carcinoma and its potential as a target for anti-metastasis therapy. Oncotarget.

[58] Chougule MB, Patel AR, Jackson T, Singh M (2011). Antitumor activity of Noscapine in combination with Doxorubicin in triple negative breast cancer. PLoS ONE.

[59] Xie T, Ren HY, Lin HQ, Mao JP, Zhu T, Wang SD, Ye ZM (2016). Sinomenine prevents metastasis of human osteosarcoma cells via S phase arrest and suppression of tumor-related neovascularization and osteolysis through the CXCR4-STAT3 pathway. Int J Oncol.

[60] Maffucci T, Piccolo E, Cumashi A, Iezzi M, Riley AM, Saiardi A, Godage HY, Rossi C, Broggini M, Iacobelli S (2005). Inhibition of the Phosphatidylinositol 3-Kinase/Akt pathway by inositol pentakisphosphate results in antiangiogenic and antitumor effects. Can Res.

[61] Schnell CR, Fdr S, Allegrini PR, O'Reilly T, McSheehy PMJ, Dartois C, Stumm M, Cozens R, Littlewood-Evans A, García-Echeverría C (2008). Effects of the dual phosphatidylinositol 3-Kinase/Mammalian target of Rapamycin Inhibitor NVP-BEZ235 on the tumor vasculature: implications for clinical imaging. Can Res.

[62] Rumman M, Jung KH, Fang Z, Yan HH, Son MK, Kim SJ, Kim J, Park JH, Lim JH, Hong S (2016). HS-173, a novel PI3K inhibitor suppresses EMT and metastasis in pancreatic cancer. Oncotarget.

