# Supplementary material for: Anti-cancer effect of palmatine through inhibition of the PI3K/AKT pathway in canine mammary gland tumor CMT-U27 cells
Source: BMC Vet Res. 2023 Oct 25;19:223. doi: 10.1186/s12917-023-03782-2 (PMC10601335; doi:10.1186/s12917-023-03782-2)
Supplement: Supplementary file 1 — Additional file 1: Figure S1. Each figure (A, B, C and D) represents the uncropped scan of the western blots shown in Fig. 3, respectively. Band images shown in Fig. 3 are marked in red box. Figure S2. Each figure (A, B, C and D) represents the uncropped scan of the western blots shown in Fig. 4, respectively. Band images shown in Fig. 4 are marked in red box. The p-PTEN in the (B) gel is lightly banded, so we used an image with increased contrast in the manuscript. [file 12917_2023_3782_MOESM1_ESM.docx]

Supplementary information

**Anti-Cancer Effect of Palmatine through Inhibition of the PI3K/AKT Pathway in canine mammary gland tumor CMT-U27 cells**

Min-Jae Yoo^1^, Jawun Choi^1^, Ye-ji Jang^1^, Sang-Youel Park^1^, and Jae-Won Seol^1,*^

^1^College of Veterinary Medicine, Jeonbuk National University, Iksan 54596, Jeollabuk-do, Republic of Korea

*Correspondence: Jae-Won Seol, jwsseol@jbnu.ac.kr

Authors’ infomation

M. J. Yoo, ymin105@naver.com;

J. Choi, jwchoi@jbnu.ac.kr;

Y. J. Jang, yejiown25@gmail.com;

S. Y. Park, sypark@chonbuk.ac.kr

**(1) Uncropped scans of the Western blots shown in Figure 3 and 4.**


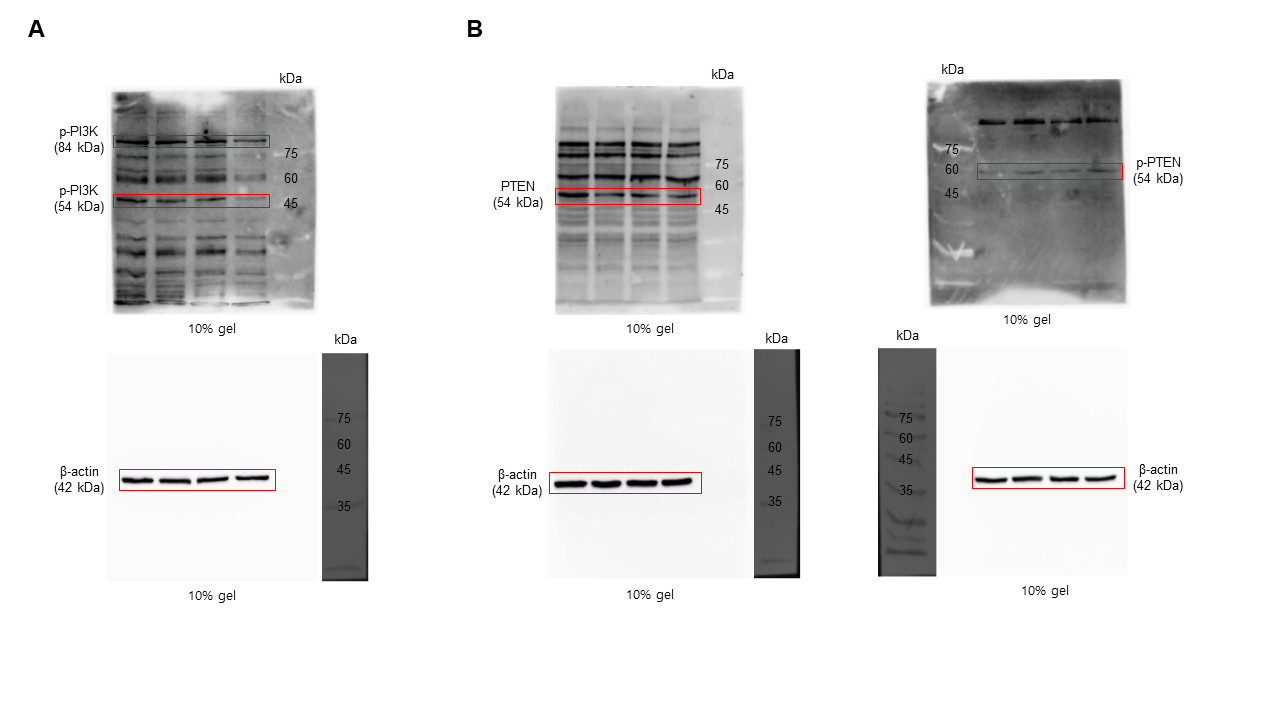

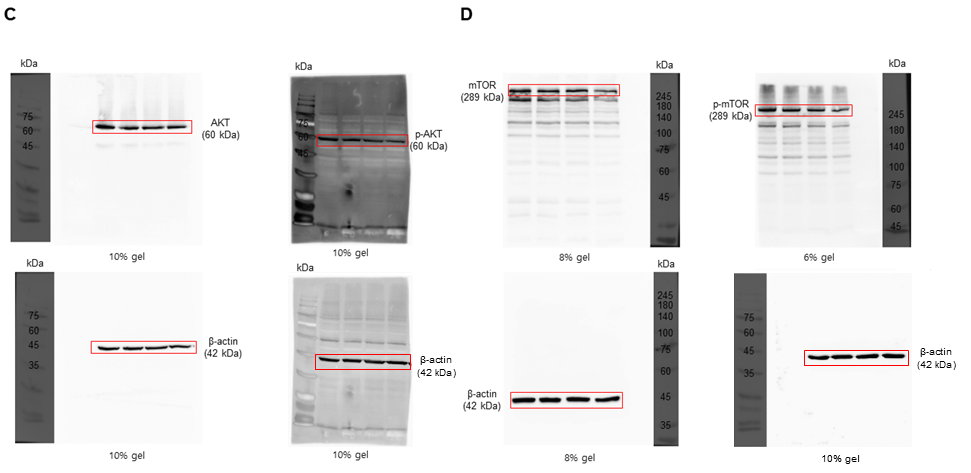


**Figure S1.** Each figure (A, B, C and D) represents the uncropped scan of the western blots shown in Figure 3, respectively. Band images shown in Figure 3 are marked in red box.


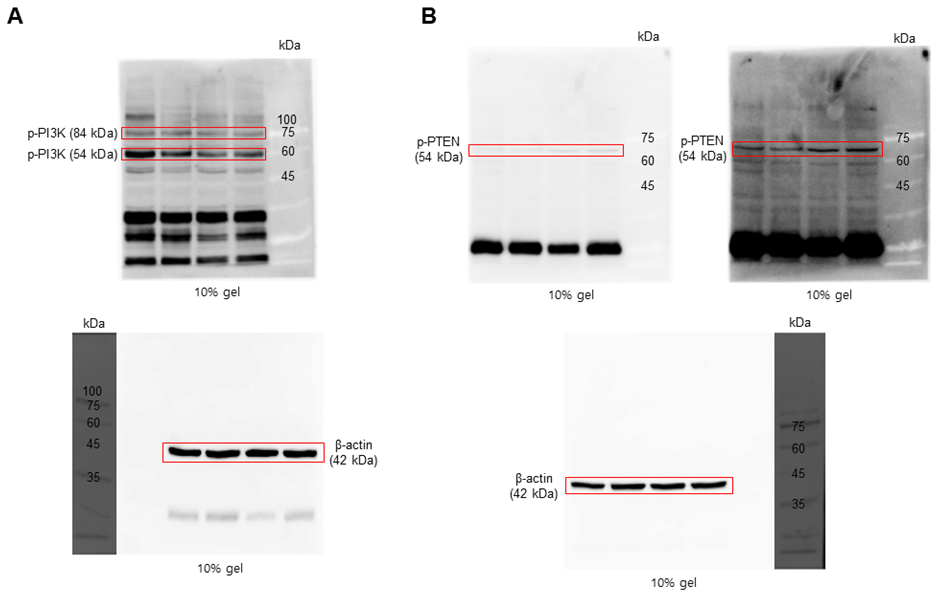

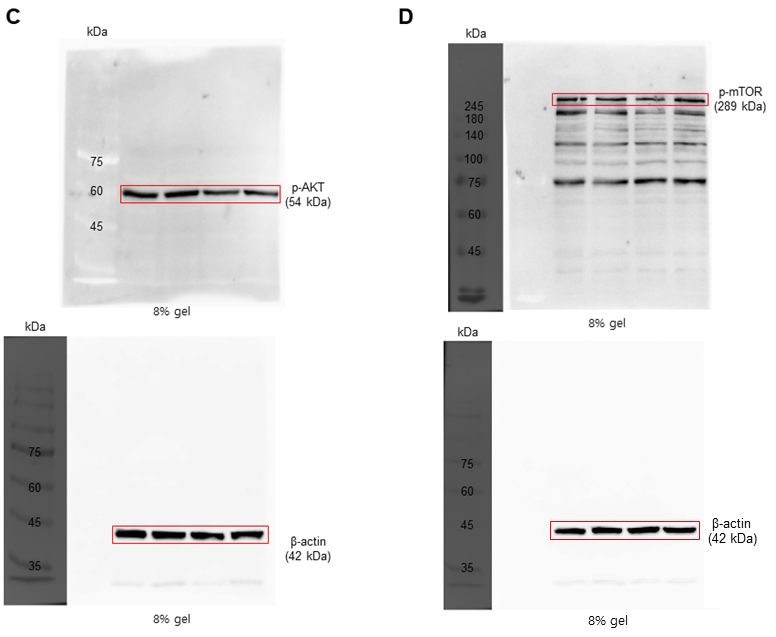


**Figure S2.** Each figure (A, B, C and D) represents the uncropped scan of the western blots shown in Figure 4, respectively. Band images shown in Figure 4 are marked in red box. The p-PTEN in the (B) gel is lightly banded, so we used an image with increased contrast in the manuscript.
